# Relative Age and Positive Youth Development in Youth Sport: Do Developmental Assets Play a Role in Creating Advantage Reversals in Female Soccer?

**DOI:** 10.3390/sports12010030

**Published:** 2024-01-12

**Authors:** Kristy L. Smith, Dennis Jackson, Patricia L. Weir

**Affiliations:** 1 Department of Kinesiology, University of Windsor, Windsor, ON N9B 3P4, Canada; weir1@uwindsor.ca; 2 Department of Psychology, University of Windsor, Windsor, ON N9B 3P4, Canada; djackson@uwindsor.ca

**Keywords:** relative age effects, developmental assets, positive youth development, advantage reversal, sport dropout, sport engagement, birth advantage, female athlete

## Abstract

Relative age effects (RAEs) are commonly associated with advantages for older athletes. However, a variety of benefits attributed to ‘advantage reversals’ have been observed among relatively younger professional athletes. Considering psychosocial development as a proposed mechanism, the purpose of this study was twofold: (1) To explore an association between developmental assets (i.e., facilitators of positive youth development [PYD]) and RAEs; (2) To assess whether overall developmental asset levels are protective against sport dropout. The Developmental Assets Profile© was distributed to members of a one-year cohort of post-adolescent, female soccer players from Ontario, Canada. The presence of differences between groups of relatively older (H1; *n* = 64) and younger (H2; *n* = 57) participants and developmental asset scales were assessed using discriminant analysis. A binary logistic regression was conducted to assess whether overall developmental asset levels are protective against sport dropout, with consideration of relevant factors. Findings suggest that relatively younger, female players score higher in two internal categories: commitment to learning and positive values. The overall developmental asset scores were not found to be protective against dropout. This study provides preliminary, albeit cautious, support that ‘advantage reversals’ may be in part associated with enhanced PYD resulting from developmental sport experiences.

## 1. Introduction

Relative age inequities are well documented in sport systems that group young participants into age-based cohorts using a designated calendar date [1,2]. While the goal of imposed age groupings is to provide developmentally appropriate training and competition for all participants [3], considerable variability in physical and psychological maturity can contribute to real or perceived performance differences at young ages through to adolescence [3,4,5,6]. These differences can lead to selection advantages and sport development opportunities (e.g., access to higher levels of competition, training, and coaching expertise [7]) for individual athletes, depending on their birthdate position with respect to the arbitrary cut-off date used by a sport system. For instance, in sport contexts involving physicality, power, and speed where peak performance occurs after maturation is complete (e.g., soccer, ice hockey), individuals who are further along on their developmental path may be more likely to be selected at young ages. Conversely, in sports that are more artistic and/or technical in nature, where peak performance is achieved earlier in the developmental process and advanced maturation presents a disadvantage (e.g., gymnastics, figure skating), an individual who is chronologically younger/less developed may be more likely to incur advantages [1]. These advantages can accumulate over the years and lead to an increased chance of being selected again in the future, resulting in a greater likelihood of advancing to the highest levels of sport achievement (e.g., professional athlete status, selection to a national team) [1]. On the opposite end of the spectrum, those who lack an advantageous birthdate position may not have equitable access to developmental opportunities, potentially contributing to negative sport experiences or dropout from sport altogether [7,8].

With respect to team sports where physical attributes (e.g., size, power, speed) typically provide a competitive benefit, relative age advantages are often conferred to those born closer to but following the organizational cut-off (i.e., relatively oldest) and disadvantage those born later in their respective cohort (i.e., relatively youngest). However, Relative Age Effects (RAEs) are probabilistic as opposed to deterministic, meaning that diverse outcomes have been observed for individuals of similar relative age [9]. Individuals born closer to but following an organizational cut-off date are not always advantaged, nor are the later born always disadvantaged. Examples of success and/or protective factors among the relatively youngest can be found in the literature. For instance, Wattie and colleagues [10] reported lower rates of injury among relatively younger male ice hockey players (age 10–15 years). At entry levels to professional ranks, Baker and Logan [11] observed that relatively younger players were chosen earlier in the National Hockey League (NHL) entry draft of junior talent between 2000 and 2005, while McCarthy, Collins, and Court [12] observed relatively younger (male) players were more likely to make a successful transition from the junior levels to senior national teams in professional rugby union and cricket.

Likewise, a variety of benefits have been reported for the relatively youngest professional athletes, such as being more likely to reach career benchmarks (e.g., 400 games played in the NHL [13]); receive higher wages (German professional soccer [14]); experience longer career duration and selection to the most elite teams (e.g., Olympic ice hockey [15]); and have representation in later career stages of professional sport (German handball [16]). Thus, the sport-related social and organizational structures that contribute to RAEs may not disadvantage all relatively younger athletes in team sports to the extent that is often presumed [17].

McCarthy and colleagues [12] proposed the term ‘advantage reversal’ to describe the advantage that is conveyed to a small number of relatively younger participants. Similar terms include the ‘Underdog Hypothesis’ [15] and ‘inverse RAEs’ [18]. The underlying mechanisms contributing to this reversal are currently speculative. Baker and Logan [11] and Schorer et al. [16] have suggested that younger players may develop superior performance/technical skills to compete with relatively older teammates, allowing these previously disadvantaged players to excel once size differences equalize following maturation. Collins and MacNamara [19] proposed that the challenges relatively younger youth encounter may provide useful or ‘structured trauma’, facilitating the development of important qualities such as mental toughness and resilience [19,20,21], which could ultimately lead to a later career advantage.

On the opposite end of the spectrum, the perceived advantages of being relatively older may actually be detrimental to the athlete’s overall well-being in the long run. Relatively older youth theoretically have greater opportunities for early specialization, referring to concentrated training and competition in one sport at the expense of other activities at young ages [22,23]. This trajectory is associated with reduced levels of physical health and an increased risk of emotional and/or physical ‘burnout’ [24,25]. Consequently, this path may also contribute to a premature end to an athlete’s career. While the association between sport withdrawal and ‘advantage reversals’ has only preliminary evidence at best, the hypotheses discussed above (e.g., [19]) could lead to a deeper understanding of relative age trends at elite levels in some sport contexts (e.g., reduced RAE magnitude at professional levels [1]) and the associated advantages for relatively younger professional athletes.

The concept of useful challenge has surfaced in the positive youth development (PYD) literature [26]. Briefly, ‘PYD’ is a strength-based perspective that views children and youth as ‘resources to be developed’ ([27], p. 20). Optimal development occurs through appropriately structured activities and leads to a range of competencies that are beneficial or protective for young people in their current circumstances and in the future [28,29,30]. Several frameworks of measuring PYD have been put forward in the literature. For example, Lerner’s ‘Five Cs’ [31] are commonly cited, recognizing character, caring, competence, confidence, and connection as desirable outcomes. Sport-specific approaches are also available, such as Petitpas’ Framework for Planning Youth Sport Programs [32], the Personal Assets Framework [33,34], and the Applied Sport-Programming Model of Positive Youth Development [35]. The frameworks share common elements, including a focus on relationships between the individual and others (e.g., with teammates, coaches, parents), and on the context in which the sport takes place (e.g., organizational structure of the sport club, characteristics of the broader community wherein participation occurs).

Benson’s 40 developmental assets [36] are believed to facilitate PYD when delivered through youth programming [29,35]. These assets have been described as the ‘building blocks’ of human development, and asset possession is believed to provide a protective, enhancement and resiliency role for youth [36]. In the realm of sport, developmental asset possession has been proposed to impact personal development, performance factors, and lifelong participation [33,34,35]. Specific links have been reported between developmental assets and sport outcomes by Strachan et al. [25], who identified an association between three developmental asset categories (positive identity, empowerment, and support as measured by the *Developmental Assets Profile* [37]) and two important sport outcomes (reduced burnout and enhanced enjoyment) in a sample of competitive adolescent athletes (*n* = 123). Developmental assets have also been differentiated at the environmental level, supporting the importance of context. Fraser-Thomas and colleagues [38] demonstrated that competitive swimmers (overall *n* = 181) from smaller communities (i.e., less than 500,000 inhabitants) scored higher on the commitment to learning, positive identity, empowerment, and support categories [37] compared to individuals from larger cities.

There has been debate as to whether positive developmental outcomes are automatically incurred as a result of sport participation (compare [29] vs. [39]). Holt and colleagues [29] synthesized the qualitative findings generated for more than 2400 individuals and concluded that sport participation can routinely lead to identifiable positive outcomes within a PYD climate, although negative findings were excluded from the meta-analysis. However, Fraser-Thomas and colleagues [35] outline in the Applied Sport-Programming Model of Positive Youth Development that if sport program delivery is not suitable for all participants (e.g., developmental assets are not being promoted, challenges are not developmentally appropriate and result in negative sport experiences), PYD may be limited, and an increased risk of dropout may ensue. Therefore, the presence or absence of development assets could promote sport engagement and facilitate PYD or, alternatively, lead to dropout and reduced PYD.

To date, direct examinations of PYD and RAEs are limited within the literature. However, relative age has been associated with measures of psychosocial well-being which share similar characteristics of existing PYD frameworks (e.g., Lerner’s ‘Five Cs’ [31]). For example, Duncan and colleagues [40] utilized Diener’s 8-item Flourishing scale [40,41] to assess feelings of competence, optimism, purpose, and success in personal relationships and reported lower scores for the later-born fourth quartile, supporting the negative impact of being relatively younger. Given that relative age can alter the impact of sport program delivery for each respective participant (i.e., the relatively oldest are provided with development opportunities while the relatively youngest are overlooked), it seems necessary to explore the relationship between developmental assets and youth sport participation. Related findings could help to unravel the ‘reversal of advantage’ for the relatively younger participants and provide insight to improve the sport experience for all athletes. Thus, the primary objective of this study was to explore the possibility of a relationship between developmental assets (i.e., facilitators of PYD) and RAEs within the realm of sport in a post-adolescent sample. It was hypothesized that relatively younger participants who maintained participation in the sport system until post-adolescence (i.e., beyond 15 years of age) may ultimately benefit from enhanced developmental asset possession as a result of the challenges encountered from being less physically and/or psychologically developed compared to peers. A secondary purpose of this study was to ascertain whether overall developmental asset levels are protective against dropout during post-adolescence (i.e., between 17 and 18 years of age), in line with implications of the Applied Sport-Programming Model of Sport Participation [35]. In doing so, this study extends the findings of Fraser-Thomas and colleagues [38] to the context of female soccer in Ontario.

## 2. Materials and Methods

### 2.1. Participants

A one-year cohort (i.e., same birth year) of female soccer participants was identified by *Ontario Soccer*, the provincial sport governing body for soccer in Ontario, Canada. Registration entries for all participants registered at age of 10 years were compiled up to and including those aged 16 years, providing a longitudinal record of participation across the adolescent transition years (for more information on participants, please refer to Smith and Weir [42]). The email addresses associated with registrants at ages 15 and 16 years (*n* = 4192) were selected for the purpose of recruiting a post-adolescent subsample who had maintained participation into the final two years under examination. An invitation to the online survey was distributed directly by the provincial organization, in order to maintain the anonymity of members. Ontario Soccer noted that email addresses provided by members could belong to the participants or to parents/guardians, and duplicate contact information (i.e., multiple contacts for the same player) may have been present in the distribution list. Instructions for survey completion were thus directed to the player, and also to the parent (i.e., to be completed by daughter(s) currently or previously registered in youth soccer) to account for instances where the provided email address did not belong to the youth participant. The first portion of the survey included 9 demographic questions, followed by the 58-item Developmental Assets Profile (DAP) [37].

### 2.2. Research Process

Prior to data cleaning, 177 individuals provided consent and started the survey. The DAP requires no more than six questions be left unanswered (corresponding to 10% missing data). Fifty-one respondents did not meet this criterion and were removed due to insufficient data. The average DAP completion time has been found to lie between five and seven minutes [43]; thus, the remaining responses were reviewed with respect to completion time and reliability. An additional four participants were removed, resulting in 68% of initial respondents being retained. The remaining sample of female participants (*n* = 121) were between the ages of 15 and 19 years (M = 17.1; SD = 0.37).

All responses were coded for relative age based on the 31 December cut-off employed by Ontario Soccer for age groupings. Sample size requirements for reporting purposes (i.e., minimum of 30 responses per group [43]) and a desire to maintain the maximal amount of statistical power dictated that half-year comparisons would be possible. Thus, all participants born in January through the end of June were coded as relatively older (H1), and those born in July through December were coded as relatively younger (H2). Further coding of demographic data was completed and summarized in Table 1.

### 2.3. Research Tool

Respondents rated the relevance of 58 items from the DAP questionnaire on a four-point scale (i.e., ‘Not at all or rarely’ = 0 to ‘Extremely or almost always’ = 3). Sample questions include ‘I tell the truth even when it is not easy’, ‘I take responsibility for what I do’, and ‘I deal with frustration in positive ways’. This questionnaire was designed to capture the developmental experiences of young people in grades six through twelve, and has been found to be a valid and reliable measure through field tests (*n* = 1300) [43,44]. Quantitative scores were calculated for eight developmental asset scales, four external (support, empowerment, boundaries and expectation, constructive use of time) and four internal (commitment to learning, positive values, social competencies, and positive identity). An outline of the eight scales is provided by the Search Institute ([43], p. 5) and summarized in Appendix A. Participants could score a maximum of 30 points on each respective asset scale. The overall external and internal asset scores were then calculated (representing the average of the four respective scales for each category and thus ranging from 0 to 30) and combined to calculate the overall developmental assets score (ranging from 0 to 60) in accordance with instructions provided in the DAP User Manual [43]. The overall developmental asset scores for the sample are provided in Table 2 according to the interpretative ranges provided in the DAP User Manual [43].

Reliability estimates (Cronbach alpha values) are presented in Table 3. In accordance with previous research and recommendations, values of 0.70 and above were considered to be reliable [45,46]. The scale ‘constructive use of time’ did not meet this criterion (α = 0.288). This asset category has been observed to have the lowest reliability estimate in field testing (overall α = 0.59 [43]), and has also been suggested to be unreliable for sport participants due to the definition and nature of this scale [25]. Specifically, it seeks to determine the presence or absence of involvement in any one of several possible enriching activities, rather than the quantity of such involvement [43]. It also has the fewest number of items of all scales measured in the DAP. Thus, the decision was made to remove this scale prior to conducting the MANOVA to prevent any detriment to statistical power. Strachan and colleagues [25] similarly removed this construct due to a low reliability value (α = 0.34) among their athlete sample (*n* = 123).

Five participants had missing information for chronological age (4.1%). Little’s MCARs test indicated that this information was missing completely at random (*p* = 0.242). Four participants (3.3%) had a permissible amount of missing information (range of one to two questions left unanswered per person) and this was accounted for when scale scores were calculated (see the Developmental Assets Profile User Manual [43] for more information). The missing data occurred on five separate DAP items with a maximum occurrence of one for each individual question and were thus considered to be missing at random.

### 2.4. Statistical Analyses

A one-factor, between-subjects multivariate analysis of variance (MANOVA) was planned to test for group differences between relatively older (H1; *n* = 64) and relatively younger (H2; *n* = 57) respondents on the eight development asset categories (as outlined above). Reliability estimates were calculated using Cronbach’s alpha. Data were examined for the presence of outliers and assessed for suitability based on the assumptions of MANOVA (i.e., multivariate normality and homogeneity of the covariance matrices) prior to conducting the analysis. The analysis was conducted using IBM SPSS Statistics 25. A statistically significant result (*p* < 0.05) was followed by a discriminant analysis to evaluate group membership for descriptive purposes. Structure coefficients greater than 0.33 (10% of overlapping variance) were considered eligible for interpretation [47]. Cross-validation was conducted using a random selection (i.e., 80%) to assess how well the discriminant function equation predicted the outcome.

To assess whether overall developmental asset levels are protective against sport dropout in female youth soccer, a binary logistic regression was planned to compare ‘dropout’ vs. ‘engaged’ participants (note: individuals who reported ‘not playing but planning to play in the future’ were excluded from this portion of the study) and the overall developmental asset scores (continuous scores ranging from 0 to 60). Participants who were 16 years of age or younger were excluded (*n* = 2) from this portion of the analyses to maintain consistency with respect to the chronological age of the targeted one-year cohort. The analysis was first conducted with all members belonging to the targeted one-year cohort (≥17 years of age; *n* = 102) and then re-analyzed with respondents who had missing data with respect to chronological age (*n* = 107) to assure no influence of these additional respondents on the model. This was followed by a second binary logistic regression analysis to extend previous findings [38] with respect to community size to female youth soccer participants in Ontario. This analysis was conducted with additional predictors in the model that may influence engagement, including chronological age (e.g., [38]), relative age (e.g., [8,42,48]), competition level (e.g., [42]), and age of initiation in soccer. Bootstrapped confidence intervals (95%) and standard errors were obtained. Residuals were examined to evaluate how well the model fit the data.

## 3. Results

### 3.1. Primary Findings—Relative Age and Developmental Assets

Standardized residuals were assessed to identify univariate outliers on the scale scores, with any score >±3 requiring further examination [49]. Extreme scores on two scales were identified for one participant: support (ZRE = −3.77, score of 0/30) and empowerment (ZRE = −4.18; score of 3/30). The model was statistically significant with or without this case (*p* < 0.05). In order to retain this participant in the sample but prevent undue influence, transformations of the raw scores were conducted by assigning each score to be one unit smaller than the next-most-extreme occurrence in the distribution [47]. Leverage values were examined to identify outliers on the predictors with scores >3p/n indicative of extreme values [49]; no such cases were identified.

Examinations of the normality and homogeneity of the covariance matrices underlying MANOVA did not reveal any substantial anomalies. Bivariate scatterplots of the dependent variables produced approximate elliptical scatterplots. Shapiro-Wilks’ test was significant in several instances. However, skewness and kurtosis values were within an acceptable range (within ±2 and ±3, respectively), and there was no evidence of platykurtosis, suggesting a minimal effect on power. No concerns were identified during a visual inspection of the distribution. Box’s Test of Equality of Covariance Matrices was not significant (*p* = 0.158), suggesting the covariance matrices were approximately equal, as required. The MANOVA was conducted with birth half as the independent variable (i.e., relatively older [H1] vs. relatively younger [H2]), and the seven remaining development asset scales as the dependent variables. Results from the MANOVA were statistically significant according to Wilks’ Λ (0.850), F (7, 113) = 2.850, *p* < 0.01. Therefore, the null hypothesis was rejected. Descriptive statistics are presented in Table 4.

The MANOVA was followed by a discriminant analysis to explore differences between the two groups. Preliminary analysis of the covariance matrices revealed that all developmental asset categories were positively related in both the relatively older and relatively younger groups. The canonical R^2^ was 0.15. The discriminant function (DF) coefficients and structure coefficients for the seven developmental asset scales can be found in Table 4. The correlations revealed that the internal asset categories ‘commitment to learning’ (0.402) and ‘positive values’ (0.366) contributed to group separation. The standardized DF coefficients suggested that ‘positive identity’ was the most important predictor to participant scores; however, it did not contribute highly to group separation. Thus, ‘positive identity’ was evaluated further as a potential suppressor variable; this scale appeared to exhibit a suppressor effect on the ‘social competencies’ scale when it was included in the model. The mean variate scores (group centroids) were 0.393 for H1 (relatively oldest) and 0.442 for H2 (relatively youngest).

### 3.2. Secondary Findings—Developmental Assets and Sport Dropout

Prior to conducting the binary logistic regression, the assumption of a linear relationship between continuous predictors (i.e., overall developmental asset score comprised of all eight developmental asset categories, chronological age, and age of initiation in soccer) and the logit of the outcome variable was assessed using the procedure outlined by Field ([45], pp. 792–797) based on recommendations from Hosmer and Lemeshow [50]. The estimation failed when chronological age was included, which was not surprising in light of the small number of participants outside of the 17-year-old category (*n* = 18). Thus, chronological age was removed. Interactions between the remaining predictors and each respective log transformation were not significant (*p* > 0.05), and thus were deemed to be suitable for analysis. The presence of multicollinearity was evaluated by an inspection of tolerance values, VIF, and variance proportions; no issues were noted. Finally, contingency tables were reviewed to ensure a sufficient number of participants in each cell for each categorical predictor (i.e., relative age categorized into birth halves [H1 and H2], dichotomous breakdown of community size at a criterion of 500,000 inhabitants, and competition level separated into recreational and competitive categories).

The preliminary binary logistic regression was run with and without participants with missing data for chronological age. There were no meaningful differences in the outcome, and therefore all participants classified as ‘dropout’ or ‘engaged’ were included (*n* = 107). The overall model χ^2^(5) = 9.863, *p* > 0.05, did not predict engagement in female youth soccer. Coefficients for each predictor included in the model are available in Table 5. Relative age was the only statistically significant predictor, with the relatively youngest (H2) observed to be 4.6 times more likely to be ‘engaged’ in youth soccer compared to the relatively oldest (H1) members of this sample (note: the original odds were reversed to facilitate interpretation—(i.e., odds of 0.217/1). Inspection of the standardized residuals revealed four participants (3.7% of cases) with scores outside of ±3. Upon closer inspection, the common characteristic of these four individuals was a ‘low’ score on the ‘constructive use of time’ scale (range: 8–10/30), with ‘fair to good’ overall developmental asset scores. An examination of leverage values identified two participants who were outliers on the predictor variables: one was the only 19-year-old in the sample and the other had listed her playing status as ‘occasional’ but was grouped with the ‘dropout’ players for classification purposes.

## 4. Discussion

### 4.1. General Findings

The present study is an exploratory examination of positive youth development (PYD) in female youth soccer players. The primary objective was to determine if an association exists between developmental asset scales (i.e., a facilitator of PYD) and relative age by birth halves. The secondary objective was to evaluate whether overall developmental asset scores were protective against ‘dropout’ in a post-adolescent age group with consideration of other potential predictors. Based on the data available, the findings suggest that relatively younger female soccer players possess higher levels of developmental assets in two internal categories, ‘commitment to learning’ and ‘positive values’, although the structure coefficients were ‘poor’ in nature [51]. These findings provide preliminary, albeit extremely cautious support for the hypothesis that ‘advantage reversals’ [12] may be in part associated with enhanced PYD resulting from developmental challenges or experiences (as suggested by Collins and MacNamara [19]). Overall developmental asset scores did not appear to be protective against sport-specific dropout in this context. However, relative age was observed to be an important factor, with relatively younger participants being greater than four times more likely to be engaged in soccer in this sample.

### 4.2. Detailed Findings—Relative Age and Developmental Assets

The ‘commitment to learning’ scale best differentiated relatively older and younger participants in this post-adolescent female sample. The items contained in this category reflect both the motivation to learn and active engagement in the learning process [43]. If a relatively younger athlete is presented with RAE-related challenges during the developmental levels of participation, a commitment to learn the technical aspects of their chosen sport could theoretically enable the individual to surpass the skill level of their relatively older counterparts who may rely more on advanced physical size. This finding could explain observations of superior motor performance in relatively younger athletes [52] and associated hypotheses [11,16], but could also support a commitment to learn psychological skills such as coping and persistence in the face of failure or adversity. Detailed research into the mechanism(s) by which ‘commitment to learning’ assists relatively younger athletes is required to make conclusions. It should also be noted that sport sampling, whereby young athletes participate in more than one type of sport, occurred with female soccer participants who had active registration with Ontario Soccer until at least 15 years of age. Thus, measures of this scale in relatively older and younger athletes who dropped out prior to age 15 and younger would be important to examine.

The ‘positive values’ scale reflects personal virtues of the individual; honesty, integrity, responsibility, and restraint are included, as well as caring about others and working for equality/social justice [43]. These qualities are highly reflective of both the ‘character’ and ‘caring’ outcomes outlined by Lerner and colleagues in their model for the integration of families, children, and civil society [31]. The finding of higher scores on this scale among the relatively younger may suggest enhanced PYD outcomes for the relatively younger, which has been theorized to be an outcome of useful or ‘structured trauma’ [19] resulting from the deferred position within a peer cohort. The virtues of the positive values scale may also reflect a proposed ‘6th C’ [27], ‘contribution’, as the eventual outcome of the other five. Indeed, the one participant who provided evidence of contribution in her survey responses (i.e., coaching youth soccer) scored in the top tertile for the positive values scale in this sample. Case studies of participants who transition to a contributive position in sport (e.g., coach, volunteer, referee) with consideration of RAE-related challenges may illuminate whether positive outcomes exist, over and beyond the advantages observed at professional levels of sport.

### 4.3. Detailed Findings—Developmental Assets and Sport Dropout

An increased risk of dropout was observed among relatively older participants at age 17 years in this sample (OR 4.6, 95% CI 1.39, 15.18), although it was noted that the number of ‘dropout’ players was small in comparison to the ‘engaged’ group. A similar finding has been reported among recreation-level female soccer players in Germany [53]. This increased risk for the relatively older deviates from earlier longitudinal findings in this cohort. Specifically, players born in first quartile (i.e., January through March) were observed to have a median survival of four years between the ages of 10 and 16 years, while all other quartiles had a median survival of three years (see Smith and Weir [42] for further discussion). This may suggest underlying, transient patterns of relative age advantage that require further investigation. Relatively older athletes theoretically have greater opportunities for early specialization in sport (e.g., selection for elite teams where they experience higher levels of training and competition [22,23]), a trajectory associated with additional negative aspects of sport such as burnout and injury (e.g., [24,25]). If relatively older athletes are leaving sport at earlier ages than their relatively younger peers, it could lend support to reducing specialized sport involvement at younger ages.

Sport engagement was not predicted by other variables in this sample, including overall developmental asset scores, community size, competition level, and age of initiation in soccer. While acknowledging that this study was exploratory in nature, it is surprising that community size did not emerge as a significant determinant. Fraser-Thomas et al. [38] found that practicing sport in a large city with a population greater than 500,000 significantly increased the risk of dropout among adolescent, competitive swimmers (OR 4.74, 95% CI 2.29–9.09). This did not appear to be the case in this sample and could possibly be attributed to undetermined, qualitative differences in the two sport contexts (e.g., individual vs. team sport, season length, training hours). However, this finding is preliminary and future longitudinal studies are needed to unravel the impact of community size in a more objective manner using alternative statistical techniques. Overall developmental asset levels were not protective against dropout for the adolescent competitive swimmers [38], mirroring the findings in the present study.

### 4.4. Future Directions

A future consideration would be to compare sport engagement/dropout to the eight developmental asset scales individually, rather than the overall score. This study has shown potential differences in internal asset categories when analysed by relative age, while Fraser-Thomas and colleagues [38] showed significant differences in two external and one internal category with respect to community size. The protective nature of developmental assets against sport-specific dropout is likely much more complex than can be observed using an overall score, and future studies should seek more detailed analyses with larger samples of participants. These studies should include relative age, community size, chronological age, and sex, along with other potential determinants when available (e.g., competition level). Individuals of varying chronological age and sex could not be recruited in sufficient numbers for this analysis due to logistical constraints; while this provides a purer sample in terms of temporal influences (e.g., similar sport structures being employed at the provincial level during development), it does not permit the evaluation of these relevant variables or detailed comparisons between groups.

The findings of this study are aligned with the Applied Sport-Programming Model of Positive Youth Development [35], to the extent that can be tested. The participants in this sample were engaged in soccer until at least 15 years of age (i.e., potentially avoiding the decline in physical activity participation that is often associated with adolescence; e.g., [54]), and more than half of the sample scored in the ‘good’ range or higher on overall developmental asset levels. However, a more detailed analysis of sport context is required at the club/organization and individual athlete levels to understand how developmental assets contribute to sport engagement. Although a framework that bridges the gap between these two lines of research (i.e., RAEs and PYD) is not currently available, a theoretical model should be incorporated whenever possible. For instance, Bronfenbrenner’s bioecological theory [55,56,57] suggests that several interacting systems play a role in development over time. Thus, recognizing individual differences, relationships between sport stakeholders (e.g., between athletes and coaches, coaches and parents), and community/environmental level contributions will be essential in future research. Qualitative analyses in the form of case studies, interviews with athletes, coaches, and parents, and document analysis of organizational philosophies would be beneficial.

With respect to relative age research in sport, it is important to remember that being required to overcome challenges as a relatively younger participant only benefits a small number of later-born athletes. Largely, birth date inequities have been tied to sport dropout among relatively younger participants [8,48], or to a lack of registration altogether at the youngest ages [58,59]. Researchers need to determine what differences exist between those who overcome relative age disadvantages and those who decline sport participation. Considerations of how youth develop within multilevel systems, as well as individual-level analyses to examine inter-quartile and intra-quartile variation in relative age outcomes, have been recommended to better understand the probabilistic advantages/disadvantages that result from RAEs [9].

According to Holt et al.’s [29] model of PYD through sport, PYD outcomes can be obtained through both implicit (e.g., everyday interactions between athletes and coaches) and explicit processes (e.g., intentional teaching of life skills and implementation of transfer activities). The implicit pathway is important as many coaches are volunteers and may prefer not to be tasked with the additional responsibilities of a life skill building program. This implicit pathway may provide an explanation for enhanced PYD outcomes in the relatively younger, should future studies continue to provide evidence of this trend.

### 4.5. Practical Implications for Sport Stakeholders

The provision of an appropriate sport climate and supportive relationships may assist relatively younger athletes in team-based contexts to overcome the sport-related challenges that they encounter as a result of their birthdate position within an age-grouped cohort. Yet, knowledge of RAE-related mechanisms can still be applied in an explicit manner to further the development of all athletes, whether they be relatively older or younger within their peer group. For instance, all athletes could be given the opportunity to experience being both relatively older (e.g., to develop leadership skills) and relatively younger (e.g., to enhance technical and/or psychological skill development) during their athlete development years. This could potentially be accomplished using the Novem System proposed by Boucher and Halliwell [60], in which a nine-month age grouping is employed per cycle/age category at the developmental level. Similarly, selection cut-off dates could be alternated from year to year to provide each athlete an opportunity to experience variable relative age positions from season to season ([61]). Opportunities to compete against individuals of similar height, weight and/or maturity (i.e., bio-banding [62]) may be another feasible intervention to improve the sport experiences of all participants, although further empirical testing is required to determine the effectiveness of all proposed solutions. Ultimately, the ‘structured trauma’ that leads to enhanced PYD should not be a coincidental outcome of RAEs, but rather intentionally and thoughtfully incorporated into sport programming for the benefit of all participants [19].

### 4.6. Strengths and Limitations

A strength of this study is the post-adolescent subsample recruited from the province-wide cohort that was followed longitudinally from the age of 10 to 16 years. Although the exact distribution by region and overall response rate is unknown due to logistical and privacy constraints, it is believed that the invitation to participate was distributed to all participants of the same age and sex in Ontario, Canada, who had registered with Ontario Soccer within the designated two-year period. The limitations of this study include concerns inherent with any type of self-report questionnaire (e.g., social desirability bias, response bias), a small sample size, and unequal group sizes (engaged > dropout). A detailed breakdown of participants beyond first and second halves of the year, dichotomous community sizes, etc., was not possible and may have resulted in a loss of information. Future studies of this nature should seek a larger number of respondents along with a more representative sample across chronological age groups, sex, and geographical regions. Furthermore, the overall developmental asset scores could not be used in the binary logistic regression without the inclusion of the constructive use of time scale, a subcategory with relatively low internal consistency and some questionability among athlete populations. Future research would also benefit from the knowledge of whether dropout in one sport is related to engagement in another sport context. This information was available for this sample of participants, but the limited sample size prevented analysis related to the magnitude of sport involvement.

## 5. Conclusions

The findings of this study provide preliminary evidence that relatively younger athletes who maintain participation status through the post-adolescent years may benefit from enhanced PYD resulting from developmental sport experiences. However, overall developmental asset scores did not appear to be protective against sport dropout in this particular sample of female soccer players.

## Figures and Tables

**Table 1:** Selected demographic information.

	Number of Participants	Percent of Sample (%)
Participant status in most recent year		
No longer playing soccer (‘dropout’)	22	18.2
Not playing but planning to play in future	11	9.1
Playing soccer (‘engaged’)	87	71.9
Other: coaching	1	0.8
		
Competition level in most recent year of participation		
Recreational (e.g., house league, ‘just for fun’)	52	43.0
Competitive (e.g., travel, representative)	69	57.0
		
Initiation age for soccer (based on Ontario Soccer LTPD)		
Active Start (U4–U5, inclusive of age 5)	83	68.6
FUNdamentals (U6–U8)	31	25.6
Learn to Train (U9–U12)	7	5.8
Soccer for Life (13+)	0	0
		
Current community size (estimated by participant)		
Not sure	3	2.5
Rural/small town (e.g., less than 5000 people)	12	9.9
Medium-sized town or city (e.g., 5000–500,000 people)	74	61.2
Large city (e.g., more than 500,000 people)	32	26.4
		
	Mean (Median)	Range
Engagement in additional sports (other than soccer)	1.81 (1)	0–8

**Table 2:** Overall developmental asset scores according to interpretative ranges.

Interpretative Ranges for the Overall Developmental Asset Scores	Number of Participants	Percent of Sample (%)
Excellent (51–60)	12	9.9
Good (41–50)	53	43.8
Fair (30–40)	50	41.3
Low (0–29)	6	5.0

**Table 3:** Internal consistency reliabilities for the Developmental Assets Profile (DAP) scales.

	Cronbach Alpha Values (α)	
Developmental Asset Scales	Present Study	Field Testing (Female Participants) Search Institute [43]
External		
Support	0.802	0.85
Empowerment	0.752	0.78
Boundaries and expectations	0.813	0.85
Constructive use of time	0.288 *	0.55
Internal		
Commitment to learning	0.720	0.83
Positive values	0.795	0.85
Social competencies	0.704	0.81
Positive identity	0.840	0.84

* Indicates low internal consistency/reliability of scale. A similar finding among athletes has been reported for ‘constructive use of time’ [25].

**Table 4:** Means (M), standard deviations (SD), discriminant function (DF) coefficients [DF without suppressor variable], and structure coefficients for relatively older (H1) and relatively younger (H2) participants on developmental asset scales.

Developmental Asset Scales	Group	M	SD	Standardized DF Coefficient	Canonical Variate Structure Coefficients
External assets:					
Support	H1	21.63	5.722	−0.275	−0.097
	H2	21.18	5.349	[−0.753]	
	Total	21.41	5.531		
Empowerment	H1	23.02	4.282	−0.611	−0.093
	H2	22.67	4.730	[−0.959]	
	Total	22.85	4.483		
Boundaries and expectations	H1	20.77	5.209	0.709	0.226
	H2	21.70	4.675	[0.995]	
	Total	21.21	4.966		
					
Internal assets:					
Commitment to learning	H1	21.56	4.642	0.653	0.402 *
	H2	23.07	4.309	[0.513]	
	Total	22.27	4.533		
Positive values	H1	19.91	4.389	0.618	0.366 *
	H2	21.21	4.135	[0.526]	
	Total	20.52	4.303		
Social competencies	H1	21.53	4.071	0.282	0.301
	H2	22.51	3.680	[0.054]	
	Total	21.99	3.906		
Positive identity	H1	18.72	5.789	−1.067	−0.171
	H2	17.91	5.485	[N/A]	
	Total	18.34	5.638		

The maximum score for each scale is 30; * indicates eligibility for interpretation [47].

**Table 5:** Coefficients of the model predicting sport engagement [95% BCa bootstrap confidence intervals based on 1000 samples].

Included	B [95% CI]	SE (B)	Odds Ratio	95% CI for Odds Ratio	
				Lower	Upper
Constant	1.117 [−3.893, 22.187]	3.765			
Overall developmental assets score	0.038 [−0.046, 0.126]	0.039	1.038	0.969	1.113
Community size [Sm.:Lg.]	0.044 [−1.780, 1.429]	1.692	1.045	0.313	3.496
Relative age [H1:H2]	−1.526 [−2.734, −0.857]	2.402	0.217 *	0.066	0.717
Competition level [Rec.:Comp.]	−0.467 [−1.605, 0.525]	0.608	0.627	0.218	1.798
Age of initiation in soccer	−0.015 [−0.379, 0.336]	0.188	0.985	0.696	1.393

Model χ^2^(5) = 9.863, *p* = 0.079; * *p* < 0.05; Hosmer and Lemeshow = 0.103; Cox and Snell = 0.090; Nagelkerke = 0.145.

## Data Availability

The data analyzed in this study were provided by Ontario Soccer. Access to the data is not available on ethical grounds as it contains personal information.

## Appendix A

### Summary of Developmental Asset Scales

**Published as: Search Institute (2005) ([43], p. 5). Developmental assets profile: User manual (p. 5). Minneapolis, MN: Search Institute.**

**External Asset Scales**
➢ Support: Support from parents, family, and other adults; parent–adolescent communication; advice and help from parents; helpful neighbours; and caring school environment.
➢ Empowerment: Feeling safe at home, at school, and in the neighbourhood; feeling valued; and having useful jobs and roles.
➢ Boundaries and expectations: Having good role models; clear rules at home and school; encouragement from parents and teachers; and monitoring by family and neighbours.
➢ Constructive use of time: Participation in religious or spiritual activity; involvement in a sport, club, or group; creative activities; and quality time at home.
**Internal Asset Scales**
➢ Commitment to learning: Enjoys reading and learning; caring about school; doing homework; and being encouraged to try new things.
➢ Positive values: Standing up for one’s beliefs; taking responsibility; avoiding alcohol, tobacco, and drugs; valuing honesty; healthy behaviours; being encouraged to help others; and helping, respecting, and serving others.
➢ Social competencies: Building friendships; properly expressing feelings; planning ahead; resisting negative peer pressure; being sensitive to and accepting others; and resolving conflicts peacefully.
➢ Positive identity: Optimism; locus of control; and self-esteem.
